# Effects of Ultrasound-Guided Erector Spinae Plane Block on Early Postoperative Inflammatory and Metabolic Parameters in Patients Undergoing Breast Cancer Surgery

**DOI:** 10.3390/life16101585

**Published:** 2026-09-22

**Authors:** Aleksandar Nikolić, Jelena Živadinović, Gorana Nedin Ranković, Dane Krtinić, Marija Stošić, Milena Stojanović, Vesna Dinić, Nikola Milenković, Nada Pejčić, Iva Binić, Milan Lazarević

**Affiliations:** 1 Department of Surgery and Anesthesiology with Reanimatology, Faculty of Medicine, University of Niš, 18000 Niš, Serbia; jelena5491@gmail.com (J.Ž.); marija91nis@gmail.com (M.S.); vesnadinic1981@gmail.com (V.D.); 2 Clinic for Anesthesia and Intensive Care, University Clinical Center Niš, 18000 Niš, Serbia; milenastojanoviclaci@gmail.com (M.S.); nada.pejcic@gmail.com (N.P.); 3 Department of Pharmacology with Toxicology, Faculty of Medicine, University of Niš, 18000 Niš, Serbia; gorana.nedin.rankovic@medfak.ni.ac.rs (G.N.R.); dane.krtinic@medfak.ni.ac.rs (D.K.); 4 Clinic of Oncology, University Clinical Center Niš, 18000 Niš, Serbia; 5 Clinic for Cardiovascular and Transplantation Surgery, University Clinical Center Niš, 18000 Niš, Serbia; milan.lazarevic@medfak.ni.ac.rs; 6 Department of Infectious Diseases and Epidemiology, Faculty of Medicine, University of Niš, 18000 Niš, Serbia; milenkovic@dr.com; 7 Clinic for Infectious Diseases, University Clinical Center Niš, 18000 Niš, Serbia; 8 Department of Psychiatry, Faculty of Medicine, University of Niš, 18000 Niš, Serbia; ivabinic@gmail.com; 9 Clinic for Psychiatry, University Clinical Center Niš, 18000 Niš, Serbia; 10 Department of Microbiology and Immunology, Faculty of Medicine, University of Niš, 18000 Niš, Serbia

**Keywords:** erector spinae plane block, early postoperative inflammatory response, metabolic parameters, breast cancer surgery, regional anesthesia

## Abstract

*Background and Objectives:* Surgical treatment of breast cancer is commonly associated with a pronounced inflammatory and metabolic stress response, which may adversely affect postoperative recovery. The erector spinae plane block (ESPB) is an ultrasound-guided regional anesthesia technique that has been suggested to influence perioperative stress responses through effective analgesia and reduced opioid requirements. This study aimed to evaluate the effects of ESPB on early postoperative inflammatory and metabolic parameters in patients undergoing breast cancer surgery. The present study represents a secondary exploratory analysis of a previously reported clinical cohort. The central hypothesis was that the addition of ESPB to general anesthesia (GA) would be associated with different early postoperative patterns of inflammatory and metabolic parameters compared with GA alone. *Materials and Methods:* This secondary exploratory analysis included 48 female patients (aged 50–65 years) scheduled for modified radical mastectomy. They were sequentially allocated in an alternating manner into two groups: one receiving only GA (*n* = 24) and the other GA with ESPB (*n* = 24). Inflammatory markers (interleukin-6, leukocyte count, neutrophil-to-lymphocyte ratio, C-reactive protein, and fibrinogen) and blood glucose levels were measured preoperatively and in the immediate postoperative period. Statistical analysis was conducted using *t*-tests, Mann–Whitney tests, and repeated measures ANOVA. *Results:* Patients who received ESPB in addition to GA had significantly lower immediate postoperative CRP levels (*p* < 0.001), while leukocyte and neutrophil counts showed significantly different patterns of change over time between the groups (time × group interaction: *p* = 0.005 and *p* = 0.003, respectively). While IL-6 levels did not differ significantly between the groups, immediate postoperative blood glucose levels were significantly lower in the ESPB group (*p* < 0.001), indicating a more favorable early postoperative glycemic profile. *Conclusions:* In patients undergoing breast cancer surgery, the addition of ESPB to general anesthesia was associated with different patterns of change in selected inflammatory parameters and lower blood glucose levels during the immediate postoperative period compared with general anesthesia alone. The present findings are limited to this early postoperative time point and do not establish a sustained anti-inflammatory or metabolic effect.

## 1. Introduction

Breast cancer is the most common malignant tumor among women worldwide, with estimates for 2022 indicating over two million new cases annually, making it a significant global health concern [1]. Surgical resection of the tumor remains a cornerstone of treatment for many patients. However, the postoperative period is often accompanied by a substantial inflammatory and metabolic stress response, which may negatively affect recovery [2].

The postoperative inflammatory response has been associated with an increased risk of complications, including infections and delayed wound healing, potentially contributing to prolonged hospital stays and a more challenging recovery process [3]. Additionally, metabolic disturbances such as hyperglycemia and insulin resistance frequently occur after surgery and may further contribute to adverse postoperative outcomes [4]. The magnitude and temporal profile of the inflammatory response vary among biomarkers; cytokines such as interleukin-6 (IL-6) may reflect relatively early changes following surgical stress, whereas acute-phase proteins such as C-reactive protein (CRP) and fibrinogen generally exhibit slower kinetics. This distinction is particularly important when interpreting biomarker changes measured in the immediate postoperative period.

Regional anesthesia techniques, including PLAN A blocks, are increasingly incorporated into multimodal analgesic strategies to reduce postoperative pain and opioid requirements. These techniques provide effective analgesia and may contribute to attenuation of the perioperative stress response, particularly in patients undergoing extensive surgical procedures such as breast cancer surgery [5,6].

Among these techniques, the erector spinae plane block (ESPB) has gained increasing attention because of its relatively straightforward ultrasound-guided application and its potential to provide analgesia across multiple thoracic dermatomes. ESPB has been used for perioperative analgesia in various surgical procedures, including breast cancer surgery [7]. Previous studies have also investigated its potential influence on perioperative inflammatory markers, including IL-6 and CRP [8,9].

Although ESPB has been increasingly investigated for perioperative analgesia and pain management, several aspects of its mechanisms and broader clinical effects remain to be fully elucidated [10]. In particular, evidence regarding its association with the early postoperative inflammatory and metabolic response remains limited. The potential effects of ESPB on perioperative changes in leukocyte and neutrophil counts, neutrophil-to-lymphocyte ratio (NLR), acute-phase reactants, and blood glucose therefore warrant further investigation.

The present study represents a secondary exploratory analysis of the same clinical cohort previously reported by our group [11]. The previous publication primarily focused on hemodynamic stability and postoperative pain, whereas the present analysis specifically evaluates inflammatory and metabolic parameters. The trial was retrospectively registered after completion of patient enrollment, and the inflammatory and metabolic endpoints evaluated in the present analysis were not listed as prespecified secondary outcomes in the trial registry. Therefore, these outcomes and the corresponding findings should be considered exploratory.

Accordingly, the aim of the present secondary exploratory analysis was to evaluate the association between ESPB and changes in selected inflammatory and metabolic parameters during the immediate postoperative period in patients undergoing breast cancer surgery.

## 2. Materials and Methods

### 2.1. Study Design

This study represents a secondary exploratory analysis of a previously investigated clinical cohort comprising 48 female patients aged 50–65 years who underwent breast cancer surgery between January and October 2023. The same clinical cohort was previously reported by our group [11], with the previous publication primarily focusing on intraoperative hemodynamic stability and postoperative pain. The present analysis specifically evaluates inflammatory and metabolic parameters that were not the primary focus of the previous publication.

The study was conducted at the Clinic for Anesthesia and Intensive Care and the Clinic for Endocrine Surgery at the University Clinical Center Niš, Serbia. The study protocol received ethical approval before patient enrollment from the Ethics Committee of the University Clinical Center Niš (Ref. 35797/4) and the Ethics Committee of the Faculty of Medicine, University of Niš. All participants provided written informed consent at least 24 h before surgery and prior to enrollment.

Patient enrollment was conducted between January and October 2023. The study was retrospectively registered in the ISRCTN registry (ISRCTN16469348) after completion of patient enrollment. The inflammatory and metabolic parameters evaluated in the present analysis were not listed as prespecified secondary outcomes in the trial registration and are therefore reported as exploratory outcomes.

The age range of 50–65 years was selected to increase the homogeneity of the study population and reduce potential interindividual variability, particularly in metabolic and inflammatory responses to surgical stress.

### 2.2. Study Groups

Patients were sequentially allocated in an alternating manner to two groups. The control group (GA) received standard general anesthesia, while the experimental group (GA + ESPB) received an ultrasound-guided erector spinae plane block (ESPB) in addition to general anesthesia, administered prior to induction. No randomization procedure was used.

Inclusion criteria were female sex, age 50–65 years, histologically confirmed breast cancer, American Society of Anesthesiologists (ASA) physical status I–II, and written informed consent for study participation. This specific age range was selected to increase the overall homogeneity of the study population and to reduce potential age-related variability in immunological and metabolic responses and comorbidities. However, menopausal status was not directly assessed and was therefore not controlled for by this age restriction.

Exclusion criteria included known allergy to any anesthetic agent used in the study, diabetes mellitus, acute infection, chronic inflammatory diseases (including autoimmune or rheumatic conditions), and long-term corticosteroid or analgesic therapy.

All included patients underwent modified radical mastectomy (MRM), defined as removal of the entire breast together with axillary lymph node dissection while preserving the pectoral muscles, for the treatment of breast cancer.

### 2.3. Technique of ESPB Administration and Anesthesia

Before induction of anesthesia, patients in the GA + ESPB group received an ultrasound-guided ESPB. During the procedure, patients were placed in a seated position on the operating table according to a predefined protocol. The block was performed using a SonoScape P10 ultrasound device (SonoScape Medical Corp., Shanghai, China), with the ultrasound probe positioned longitudinally at the level of the T4 transverse process, approximately 3 cm lateral to the midline.

The T4 transverse process and the erector spinae muscle (ESM) were used as ultrasound landmarks. Under aseptic conditions, a UPB 50 block needle (22G, 0.70 mm × 50 mm; Temena Group, Felsberg, Germany) was inserted at a 30–45° angle in a cranio-caudal direction until the needle tip contacted the T4 transverse process. Hydrodissection with 2 mL of isotonic saline was performed to confirm correct needle placement. Subsequently, 30 mL of 0.25% levobupivacaine (Levobupivacaine, Fresenius Kabi, Bad Homburg, Germany) was injected into the fascial plane between the ESM and the T4 transverse process.

Successful administration of the block was confirmed by ultrasound visualization of an anechoic fluid collection separating the ESM from the T4 transverse process (Figure 1).

In both groups, general anesthesia was administered using target-controlled infusion (TCI) of propofol (Propofol^®^ 1%, Fresenius Kabi, Germany, Bad Homburg, Germany) and remifentanil (Remifentanil B. Braun, B. Braun Melsungen AG, Melsungen, Germany During induction, propofol was administered at a target concentration of 5 µg/mL, which was subsequently reduced to 2–3 µg/mL for maintenance. At the end of surgery, the target propofol concentration was further reduced to 0.5 µg/mL to facilitate gradual emergence from anesthesia. Remifentanil was administered according to the Minto model, with an initial target concentration of 6 ng/mL during induction and subsequent adjustment to 3–5 ng/mL throughout the surgical procedure.

For postoperative analgesia, 1 g of paracetamol and 30 mg of ketorolac were administered 25 min before the end of surgery. Remifentanil infusion was discontinued 5 min before emergence from anesthesia. Rocuronium bromide was used to facilitate tracheal intubation and maintain neuromuscular blockade, with an initial dose of 0.6 mg/kg followed by bolus doses of 0.15 mg/kg as required throughout the procedure. At the end of surgery, residual neuromuscular blockade was reversed using 0.035 mg/kg neostigmine and 0.015 mg/kg atropine.

Mechanical ventilation was provided using a Dräger Perseus^®^ anesthesia workstation (Drägerwerk AG & Co., Lübeck, Germany) throughout the surgical procedure. Alaris™ PK Plus MK4 infusion pumps (CareFusion, Basingstoke, Hampshire, UK) were used for TCI.

Intraoperative monitoring included continuous electrocardiography (leads II and V5), non-invasive blood pressure monitoring, and pulse oximetry.

All modified radical mastectomies were performed by the same experienced surgical team to maintain procedural consistency. All ESPBs were performed by the same anesthesiologist according to the standardized technique described above. This approach was intended to reduce procedural variability between patients.

### 2.4. Outcomes

The inflammatory and metabolic parameters evaluated in the present secondary analysis were considered exploratory outcomes, as they were not listed as prespecified secondary outcomes in the trial registration. The inflammatory response to surgical stress was assessed using interleukin-6 (IL-6), leukocyte count, neutrophil-to-lymphocyte ratio (NLR), C-reactive protein (CRP), and fibrinogen. Blood glucose concentration was assessed as a marker of the metabolic response to surgical stress.

Postoperative pain was assessed using the visual analog scale (VAS) at predefined intervals during the first 24 h after surgery. Pain and hemodynamic outcomes were the primary focus of our previous publication based on the same clinical cohort [11] and are therefore not presented in detail in the current manuscript. The present secondary exploratory analysis focuses specifically on inflammatory and metabolic parameters.

### 2.5. Outcome Measurement

Each parameter was measured at two time points. The first blood sample was collected before induction of anesthesia, immediately after intravenous cannulation. The second blood sample was collected in the recovery room 10 min after awakening following completion of surgery and therefore represents the immediate postoperative measurement.

Leukocyte, neutrophil, and lymphocyte counts were determined using an ABX Pentra XLR 220V “EN” hematology analyzer (HORIBA ABX SAS, Montpellier, France). The laboratory reference range for leukocyte count was 4.0–9.0 × 10^9^/L. The neutrophil-to-lymphocyte ratio (NLR) was calculated by dividing the absolute neutrophil count by the absolute lymphocyte count.

Serum interleukin-6 (IL-6) concentrations were determined using an electrochemiluminescence immunoassay (ECLIA; Elecsys IL-6, REF 05109442190, Roche Diagnostics) on a Cobas e411 analyzer (Roche Diagnostics GmbH, Mannheim, Germany). According to the manufacturer’s specifications for the reagent used during the study period, the limit of detection (LOD) was 1.5 pg/mL, and the measuring range was 1.5–5000 pg/mL; a separate limit of quantification (LOQ) was not specified. Values below the LOD were reported by the analyzer and laboratory information system as “<1.5 pg/mL”, without assignment of an exact numerical concentration. Because these values were not available as exact numerical concentrations, IL-6 results were analyzed categorically as concentrations below versus at or above the LOD.

C-reactive protein (CRP) and blood glucose concentrations were measured using standardized methods according to the International Federation of Clinical Chemistry and Laboratory Medicine (IFCC) on a Beckman Coulter/Olympus AU680 biochemical analyzer (Beckman Coulter, Brea, CA, USA). The laboratory reference ranges were 0.0–5.0 mg/L for CRP and 3.9–6.1 mmol/L for blood glucose.

Fibrinogen concentration was determined using the Clauss method on a BE Trombostat coagulation analyzer (Behnk Elektronik, Norderstedt, Germany). The laboratory reference range for fibrinogen was 2.0–4.0 g/L.

### 2.6. Sample Size Calculation

The sample size calculation was performed using G*Power version 3.1.9.2 (Heinrich Heine University Düsseldorf, Düsseldorf, Germany). Based on previous research [12], with a significance level of 0.05 and a statistical power of 95%, the minimum required sample size was estimated to be 26 participants (13 patients per group). To account for potential participant loss, a total of 48 patients were included, with 24 patients in each group.

### 2.7. Statistical Analysis

Data were analyzed using SPSS version 16.0 (SPSS Inc., Chicago, IL, USA). Continuous variables are presented as mean ± standard deviation (SD), while categorical variables are presented as absolute numbers and percentages. Normality of data distribution was assessed using the Kolmogorov–Smirnov test. Continuous variables were compared between groups using the independent-samples *t*-test when normally distributed and the Mann–Whitney U test when the assumption of normality was not met. Categorical variables were compared using the χ^2^ test.

Repeated-measures analysis of variance (ANOVA) was used to analyze changes in inflammatory and metabolic parameters over time, considering the main effects of time and group, as well as the time × group interaction. When a statistically significant time × group interaction was identified, simple effects were examined, including comparisons between groups at individual time points and changes over time within each group. Statistical significance was set at *p* < 0.05.

## 3. Results

The study included 48 female patients who underwent modified radical mastectomy with axillary lymph node dissection for breast cancer at the Clinic for Endocrine and Breast Surgery, University Clinical Center Niš, between January and October 2023. The mean age of the patients was 59.73 ± 6.18 years (range, 50–65 years). The mean age was 60.96 ± 5.51 years in the GA group and 58.50 ± 6.68 years in the GA + ESPB group, with no statistically significant difference between the groups (*p* = 0.171).

The mean body mass index (BMI) was 24.54 ± 1.93 kg/m^2^ in the GA group and 24.17 ± 1.49 kg/m^2^ in the GA + ESPB group, with no statistically significant difference between the groups (*p* = 0.456). The distribution of ASA physical status was also comparable between the groups (*p* = 1.000). Baseline demographic and clinical characteristics are presented in Table 1.

For IL-6, concentrations below the assay limit of detection (<1.5 pg/mL) were observed in 83.3% of patients in the GA group and in 100% of patients in the GA + ESPB group. The difference between the groups was not statistically significant (*p* = 0.109).

Repeated-measures analysis of leukocyte counts revealed a statistically significant time × group interaction (*p* = 0.005). Leukocyte counts did not differ significantly between the groups either preoperatively (*p* = 0.672) or immediately postoperatively (*p* = 0.069). Within the GA group, leukocyte counts increased significantly from the preoperative to the immediate postoperative measurement (*p* = 0.011), whereas no statistically significant change was observed in the GA + ESPB group (*p* = 0.577) (Figure 2).

The analysis of neutrophil counts revealed a statistically significant time × group interaction (*p* = 0.003). Neutrophil counts did not differ significantly between the groups either preoperatively (*p* = 0.992) or immediately postoperatively (*p* = 0.068). Within the GA group, neutrophil counts increased significantly from the preoperative to the immediate postoperative measurement (*p* = 0.002), whereas no statistically significant change was observed in the GA + ESPB group (*p* = 0.700) (Figure 3).

The analysis of NLR values revealed a statistically significant time × group interaction (*p* = 0.008). Preoperatively, NLR values differed significantly between the groups (*p* = 0.011), whereas no statistically significant between-group difference was observed during the immediate postoperative period (*p* = 0.756). NLR values increased significantly from the preoperative to the immediate postoperative measurement in both groups (GA: *p* < 0.001; GA + ESPB: *p* = 0.005) (Figure 4).

CRP levels demonstrated a statistically significant time × group interaction (*p* < 0.001). Preoperative CRP levels did not differ significantly between the groups (*p* = 0.695). During the immediate postoperative period, CRP levels were significantly higher in the GA group than in the GA + ESPB group (*p* < 0.001). Within the GA + ESPB group, CRP levels decreased significantly from the preoperative to the immediate postoperative measurement (*p* = 0.018) (Figure 5).

The analysis of fibrinogen levels revealed a statistically significant time × group interaction (*p* = 0.030). Preoperatively, fibrinogen levels differed significantly between the groups (*p* = 0.048), whereas no statistically significant between-group difference was observed during the immediate postoperative period (*p* = 0.549) (Figure 6).

The analysis of blood glucose levels revealed a statistically significant time × group interaction (*p* < 0.001). During the immediate postoperative period, blood glucose levels were significantly higher in the GA group than in the GA + ESPB group (*p* < 0.001). Within the GA group, blood glucose levels increased significantly from the preoperative to the immediate postoperative measurement (*p* = 0.001), whereas no statistically significant change was observed in the GA + ESPB group (*p* = 0.197) (Figure 7).

## 4. Discussion

The present secondary exploratory analysis of a previously reported clinical cohort evaluated inflammatory and metabolic parameters during the immediate postoperative period in patients undergoing breast cancer surgery. Significant time × group interactions were observed for leukocyte count, neutrophil count, NLR, CRP, fibrinogen, and blood glucose. Leukocyte and neutrophil counts increased significantly from the preoperative to the immediate postoperative measurement in the GA group, whereas no significant changes were observed in the GA + ESPB group. CRP and blood glucose levels were significantly lower in the GA + ESPB group during the immediate postoperative period. NLR increased significantly in both groups, with a significant baseline difference between groups, while no statistically significant difference in IL-6 was observed between the groups. Fibrinogen levels also differed significantly at baseline, whereas no significant between-group difference was observed during the immediate postoperative period. Overall, these findings indicate differences in the immediate postoperative changes in the selected inflammatory and metabolic parameters between groups; however, given the exploratory nature of this secondary analysis and the single immediate postoperative sampling point, they should not be interpreted as evidence of a sustained anti-inflammatory or metabolic effect of ESPB.

Postoperative pain outcomes from this same clinical cohort have been reported previously [11] and are not the focus of the present secondary analysis. In that publication, the addition of ESPB to general anesthesia was associated with lower postoperative pain scores and reduced analgesic requirements, providing relevant clinical context for the inflammatory and metabolic parameters explored in the present analysis. Regional anesthesia techniques, including interfascial plane blocks such as PECS I, PECS II, and ESPB, are increasingly used as components of multimodal analgesia in breast surgery. PECS blocks provide analgesia to the anterior chest wall and may reduce perioperative opioid requirements, whereas ESPB is performed as a single-injection interfascial technique and may provide analgesia across multiple thoracic dermatomes [13,14]. Ultrasound guidance facilitates visualization of the relevant anatomical structures and local anesthetic spread during these procedures [15]. In the present cohort, no complications or technical difficulties related to ESPB were observed.

ESPB is an interfascial plane block that can be performed at the T4–T5 level for breast surgery. By depositing local anesthetic deep to the erector spinae muscle, the technique may provide analgesia across multiple thoracic dermatomes. One practical feature of ESPB is that it can be performed as a single-injection technique, whereas other interfascial approaches, such as combined PECS I and PECS II blocks, involve injections in different fascial planes [7,16]. The precise mechanism underlying ESPB analgesia remains incompletely understood; proposed mechanisms include the spread of local anesthetic toward the paravertebral and, in some cases, epidural spaces, potentially contributing to multilevel thoracic analgesia [17,18,19]. In our previous publication based on the same clinical cohort, the analgesic and hemodynamic outcomes associated with ESPB were reported in detail [11]. These previously reported findings provide clinical context for the present secondary exploratory analysis of inflammatory and metabolic parameters.

Effective pain control plays an important role in modulating the physiological response to surgical trauma. Inadequately controlled pain may enhance activation of the hypothalamic–pituitary–adrenal axis and the sympathetic nervous system, contributing to changes in pro-inflammatory mediators such as IL-6 and CRP [20,21]. Regional anesthetic techniques provide analgesia and may attenuate components of the inflammatory and neuroendocrine stress response to surgery [22]. Furthermore, reduced systemic opioid requirements may be relevant because opioids have been associated with immunomodulatory and immunosuppressive effects during the perioperative period [23]. These mechanisms provide a biological rationale for investigating whether ESPB is associated with changes in inflammatory and metabolic responses to surgical stress.

IL-6 is an early inflammatory mediator that may increase in response to tissue injury and has therefore been investigated as a marker of the acute systemic inflammatory response. Karakaya et al. reported elevated IL-6 levels in patients with trauma involving lower-extremity bone fractures, supporting the association between IL-6 and the acute inflammatory response to tissue injury [24].

In the present analysis, IL-6 concentrations <1.5 pg/mL were observed in 83.3% of patients in the GA group and in all patients in the GA + ESPB group. However, the difference between the groups was not statistically significant (*p* = 0.109), and therefore, these findings do not provide evidence of a differential effect of ESPB on IL-6 levels. Moreover, because IL-6 was assessed only during the immediate postoperative period, no conclusions can be drawn regarding subsequent IL-6 dynamics or sustained inflammatory effects.

Leukocytes are key components of the immune system, and changes in leukocyte count may occur in response to infection, inflammation, trauma, and physiological stress. During the perioperative period, surgical trauma can induce an acute stress response accompanied by changes in circulating leukocyte counts [25]. In the present analysis, a significant time × group interaction was observed for leukocyte count. Leukocyte counts increased significantly from the preoperative to the immediate postoperative measurement in the GA group, whereas no significant change was observed in the GA + ESPB group. This different pattern of immediate postoperative leukocyte response may reflect differences in the acute response to surgical stress between the groups; however, it should not be interpreted as evidence of a sustained anti-inflammatory effect of ESPB. Our findings are consistent with those of Aosasa et al., who reported an increase in leukocyte count immediately after surgical intervention compared with preoperative levels [26]. Further studies with serial postoperative measurements are needed to determine the temporal pattern and clinical significance of these changes.

In the present analysis, a significant time × group interaction was observed for neutrophil count. No statistically significant differences between the groups were observed either preoperatively or during the immediate postoperative period. However, neutrophil counts increased significantly from the preoperative to the immediate postoperative measurement in the GA group, whereas no significant change was observed in the GA + ESPB group. This different pattern of neutrophil response may reflect differences in the acute response to surgical stress between the groups. However, given the exploratory nature of the analysis and the single immediate postoperative measurement, these findings do not establish that ESPB directly modulates neutrophil activity or produces a sustained anti-inflammatory effect. Further studies incorporating serial postoperative measurements are needed to clarify the temporal pattern and potential clinical significance of these findings.

In the present analysis, NLR was evaluated as a marker of systemic inflammation during the perioperative period. A significant time × group interaction was observed for NLR values. However, NLR differed significantly between the groups at baseline, whereas no statistically significant between-group difference was observed during the immediate postoperative period. Furthermore, NLR increased significantly from the preoperative to the immediate postoperative measurement in both groups. Therefore, the significant time × group interaction should be interpreted cautiously, particularly in view of the baseline difference between the groups.

NLR increased significantly from the preoperative to the immediate postoperative measurement in both groups, indicating that an acute change in this inflammatory marker occurred following surgery irrespective of the anesthetic approach. In the broader oncological context, elevated NLR has been investigated as a prognostic marker in patients with breast cancer. Chen et al., in a meta-analysis including 4293 patients with breast cancer, reported an association between elevated NLR and poorer survival outcomes [27]. Similarly, Templeton et al., in a large meta-analysis of 100 studies including 40,559 patients with solid tumors, reported an association between elevated NLR and adverse survival outcomes [28]. However, these prognostic findings relate to oncological outcomes and should not be directly extrapolated to the acute perioperative changes observed in the present study. Given that NLR increased significantly in both groups and differed between the groups at baseline, our findings do not demonstrate a clear effect of ESPB on the immediate postoperative NLR response.

In the present analysis, a significant time × group interaction was observed for CRP levels. Preoperative CRP levels did not differ significantly between the groups. During the immediate postoperative period, CRP levels were significantly lower in the GA + ESPB group than in the GA group. Within the GA + ESPB group, CRP levels decreased significantly from the preoperative to the immediate postoperative measurement. However, given the kinetics of CRP and the very early timing of postoperative blood sampling, these immediate postoperative differences should be interpreted cautiously and cannot be considered evidence of a sustained anti-inflammatory effect of ESPB.

Elevated CRP levels are commonly observed following surgical procedures as part of the postoperative inflammatory response [29]. Previous research has suggested that the addition of ESPB to general anesthesia may be associated with attenuation of postoperative inflammatory responses [30]. In the present study, differences in CRP levels were observed between the groups during the immediate postoperative period. However, because CRP has slower response kinetics and the postoperative sample was obtained shortly after awakening, these findings should not be interpreted as evidence that ESPB reduces postoperative systemic inflammation or the risk of postoperative complications. Rather, our findings should be considered exploratory and warrant further investigation using serial postoperative measurements over a longer follow-up period to determine whether ESPB is associated with sustained changes in inflammatory markers.

Fibrinogen analysis revealed a significant time × group interaction. However, fibrinogen levels differed significantly between the groups at baseline, whereas no statistically significant between-group difference was observed during the immediate postoperative period. The presence of a baseline difference limits the interpretation of the observed time × group interaction and precludes attributing the subsequent pattern of fibrinogen changes specifically to ESPB. Furthermore, given the immediate postoperative timing of blood sampling, these findings do not allow conclusions regarding subsequent fibrinogen dynamics or their potential clinical significance. Further studies with serial postoperative measurements are needed to clarify whether ESPB is associated with changes in fibrinogen levels over time.

Analysis of blood glucose levels revealed a significant time × group interaction. Preoperative blood glucose levels did not differ significantly between the groups. During the immediate postoperative period, blood glucose levels were significantly higher in the GA group than in the GA + ESPB group. Furthermore, blood glucose levels increased significantly from the preoperative to the immediate postoperative measurement in the GA group, whereas no statistically significant change was observed in the GA + ESPB group. These findings indicate a different pattern of immediate postoperative glucose response between the groups; however, they should not be interpreted as evidence of sustained improvement in glycemic control.

Surgical trauma activates the neuroendocrine stress response, including the release of catecholamines and cortisol, which may contribute to perioperative hyperglycemia [31]. Stress-related hyperglycemia has also been associated with adverse postoperative outcomes, including higher complication rates and prolonged hospitalization [32]. Therefore, the observed differences in immediate postoperative glucose response should be considered exploratory and require confirmation in studies with serial postoperative measurements.

Previous studies using epidural anesthesia or analgesia provide additional context for the metabolic findings observed in the present study. In gynecological surgery, epidural anesthesia combined with general anesthesia was associated with a smaller perioperative increase in blood glucose levels compared with general anesthesia alone [33]. Similarly, epidural analgesia administered before induction of general anesthesia in elective lower abdominal surgery was reported to attenuate the hyperglycemic response to surgical stress [34]. Although these findings are consistent with the different pattern of immediate postoperative glucose response observed between the groups in our study, they cannot be directly extrapolated to ESPB because of differences in regional anesthetic technique, surgical procedures, and study populations.

Regarding inflammatory parameters, significant increases in leukocyte and neutrophil counts were observed from the preoperative to the immediate postoperative measurement in the GA group, whereas no significant changes were observed in the GA + ESPB group. These findings demonstrate a different pattern of immediate postoperative leukocyte and neutrophil responses between the groups. However, they do not establish that ESPB directly modulates immune function or reduces systemic inflammation, particularly given the exploratory nature of the analysis and the absence of serial postoperative measurements.

NLR increased significantly from the preoperative to the immediate postoperative measurement in both groups. Although a significant time × group interaction was observed, NLR differed significantly between the groups at baseline, while no significant between-group difference was observed during the immediate postoperative period. Therefore, the NLR findings should be interpreted cautiously and do not demonstrate a clear effect of ESPB on the immediate postoperative inflammatory response. CRP levels were significantly lower in the GA + ESPB group during the immediate postoperative period; however, given the early timing of blood sampling and the kinetics of CRP, this finding cannot be interpreted as evidence of a sustained anti-inflammatory effect. Similarly, fibrinogen showed a significant time × group interaction, but the presence of a significant baseline difference and the absence of a postoperative between-group difference limit conclusions regarding an effect of ESPB on this parameter.

Blood glucose levels increased significantly from the preoperative to the immediate postoperative measurement in the GA group, whereas no significant change was observed in the GA + ESPB group, and immediate postoperative glucose levels were significantly lower in the GA + ESPB group. These findings indicate differences in the immediate postoperative glucose response between the groups. However, taken together, the observed inflammatory and metabolic changes should be considered exploratory and do not establish that ESPB directly modulates systemic inflammation, immune function, or metabolic homeostasis.

One possible mechanism underlying the observed differences may involve improved analgesia and reduced perioperative opioid requirements. Previous studies have reported that ESPB can provide effective postoperative analgesia and reduce opioid consumption following surgery [35,36]. Surgical stress and opioid exposure have also been associated with alterations in perioperative immune function, which has generated particular interest in the oncological setting [37]. However, immune function, postoperative infections, and oncological outcomes were not directly assessed in the present study, and no conclusions regarding these outcomes can therefore be drawn from our findings. Postoperative pain and analgesic outcomes from this same clinical cohort have been reported previously in detail [11], whereas the present secondary exploratory analysis focuses specifically on inflammatory and metabolic parameters. Further prospective studies with larger sample sizes and serial postoperative measurements are needed to clarify whether the observed immediate postoperative differences are sustained and clinically relevant.

An additional consideration is the biological heterogeneity of breast cancer. Although the inclusion of women aged 50–65 years was intended to increase the overall homogeneity of the study population, age restriction alone cannot account for differences in menopausal status or tumor biology. Menopausal status, molecular subtype, ER/PR/HER2 receptor status, and tumor genomic characteristics were not incorporated into the present analysis. Previous evidence has demonstrated menopausal-status-associated differences in the expression of frequently mutated breast cancer genes, with several of these differences also related to ER and PR status [38]. Therefore, unmeasured patient- and tumor-related biological characteristics may have contributed to variability in the observed inflammatory and metabolic biomarker patterns and should be considered when interpreting the present findings.

This study has several limitations. First, the relatively small sample size and single-center design may limit the statistical precision and generalizability of the findings. Second, the present study represents a secondary exploratory analysis of a previously reported clinical cohort [11]. Although ethical approval was obtained before patient enrollment, the trial was retrospectively registered after completion of enrollment, and the inflammatory and metabolic parameters evaluated in the present analysis were not listed as prespecified secondary outcomes in the trial registry. Accordingly, these findings should be considered exploratory and require confirmation in prospective studies. Third, patients were allocated sequentially in an alternating manner rather than through a randomized allocation procedure, which may have introduced selection bias and resulted in baseline imbalances between the groups. In particular, significant baseline differences were observed for NLR and fibrinogen, which limit the interpretation of the corresponding time × group interactions. In addition, menopausal status and relevant tumor characteristics, including molecular subtype and ER/PR/HER2 receptor status, were not incorporated into the analysis. Restriction of the study population to women aged 50–65 years cannot fully account for this biological heterogeneity. These unmeasured factors may represent residual sources of variability in inflammatory and metabolic biomarker responses and may further limit the generalizability of the findings. Finally, blood samples were collected only before surgery and during the immediate postoperative period, 10 min after awakening. This very early postoperative sampling point, particularly considering the different kinetics of the evaluated inflammatory markers, limits conclusions regarding the subsequent evolution or persistence of the observed changes. Serial measurements at later postoperative time points, including the first postoperative day, would be necessary to determine whether these differences persist and whether they have clinical significance.

## 5. Conclusions

In this secondary exploratory analysis, the addition of ESPB to general anesthesia was associated with differences in the immediate postoperative changes of selected inflammatory and metabolic parameters in patients undergoing breast cancer surgery. In particular, different patterns of leukocyte, neutrophil, CRP, and blood glucose responses were observed between the groups. However, given the exploratory nature of the analysis and the single immediate postoperative sampling point, these findings do not establish a sustained anti-inflammatory or metabolic effect of ESPB. Further prospective studies with larger sample sizes and serial postoperative measurements are needed to confirm these findings and determine their clinical significance.

## Figures and Tables

**Figure 1:**
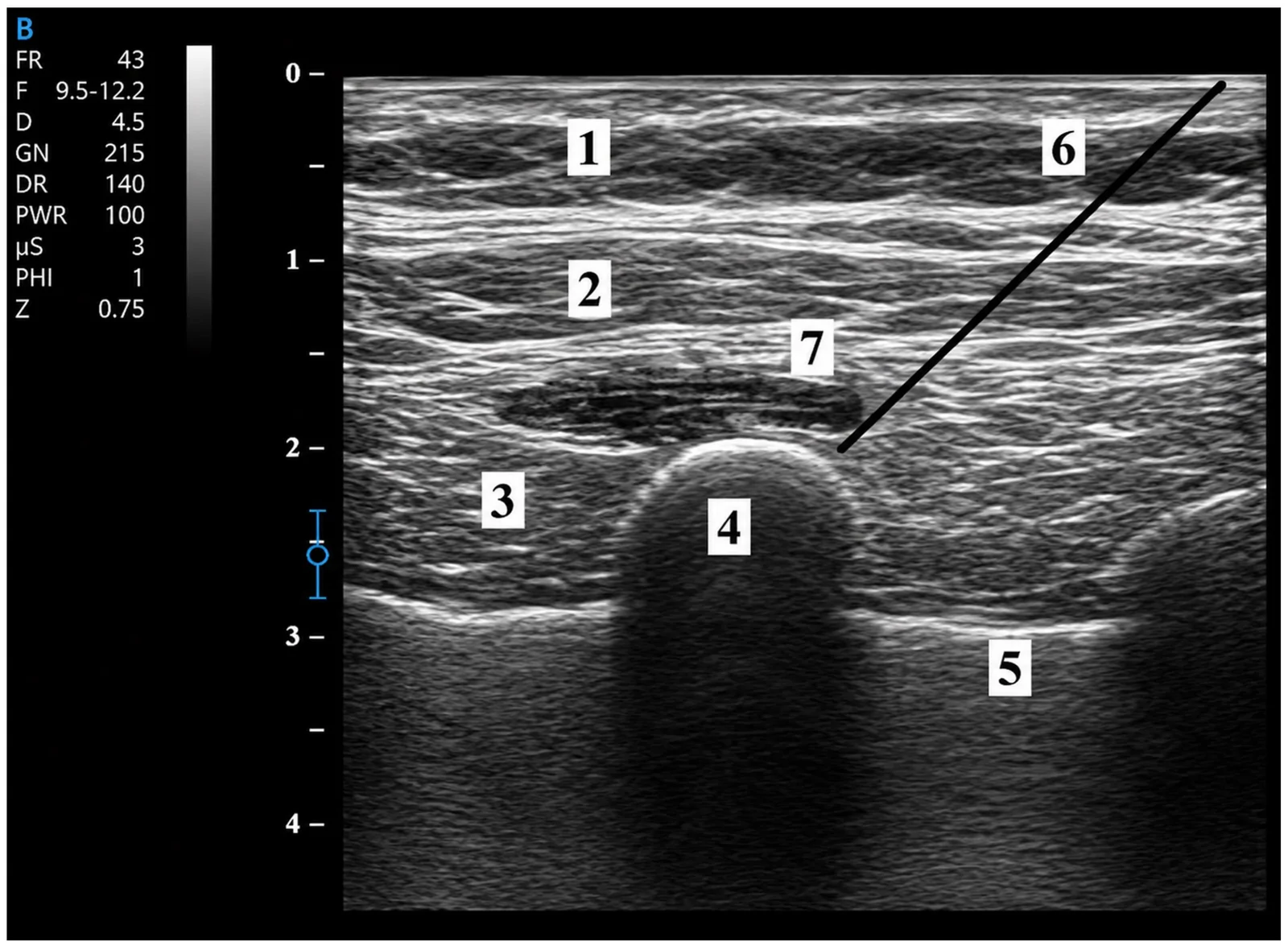
Ultrasound-guided thoracic erector spinae plane block (ESPB) at the T4–T5 level with the ultrasound probe in a longitudinal orientation. The numbered structures indicate (1) trapezius muscle; (2) rhomboid muscle; (3) erector spinae muscle (ESM); (4) transverse process and intercostal muscles; (5) pleura; (6) needle trajectory and position for ESPB; and (7) local anesthetic spread, visualized as a hypoechoic area between the ESM and the transverse process. The black line indicates the needle trajectory, with its distal end indicating the needle tip.

**Figure 2:**
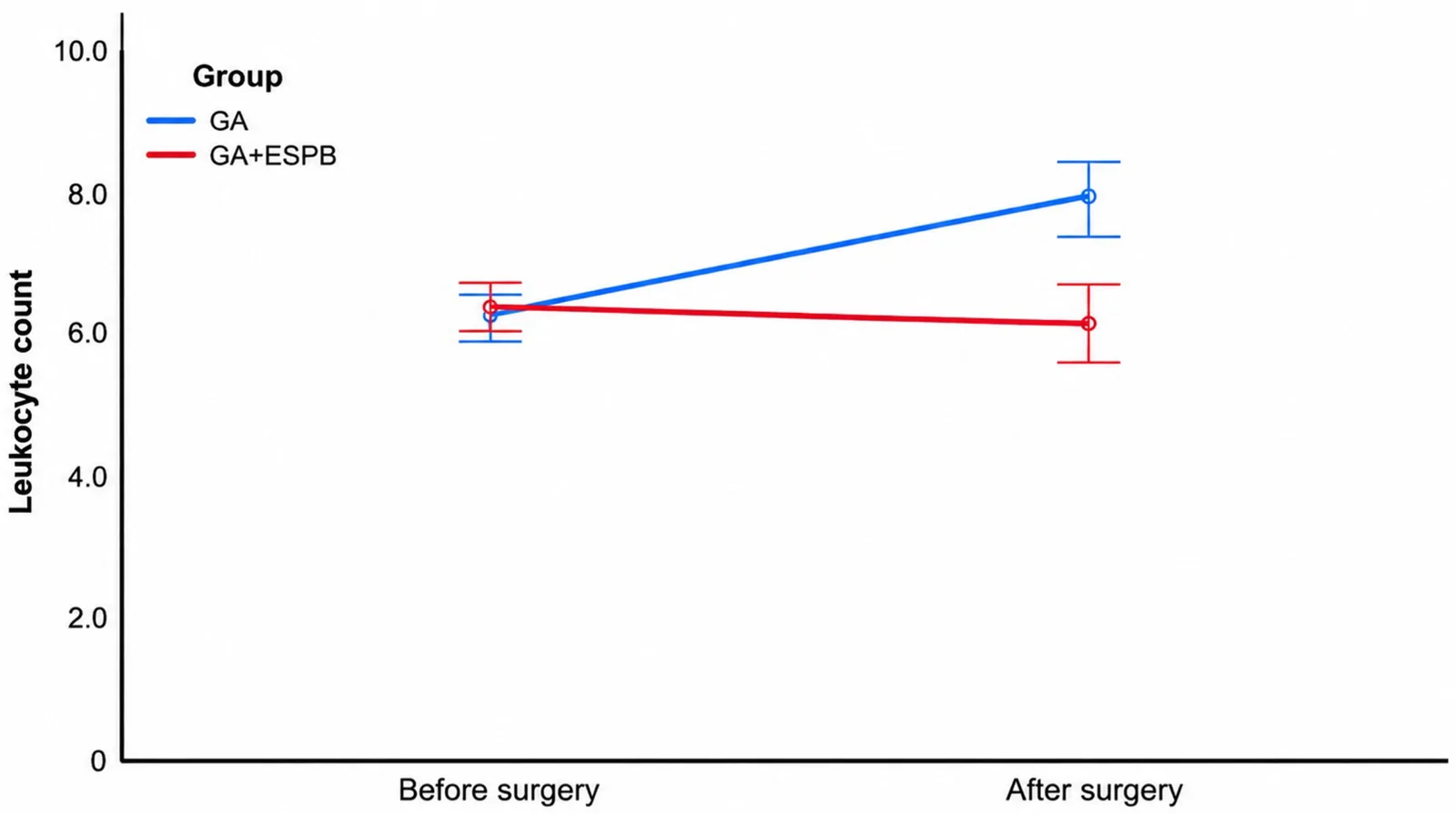
Leukocyte counts before surgery and during the immediate postoperative period in the GA and GA + ESPB groups.

**Figure 3:**
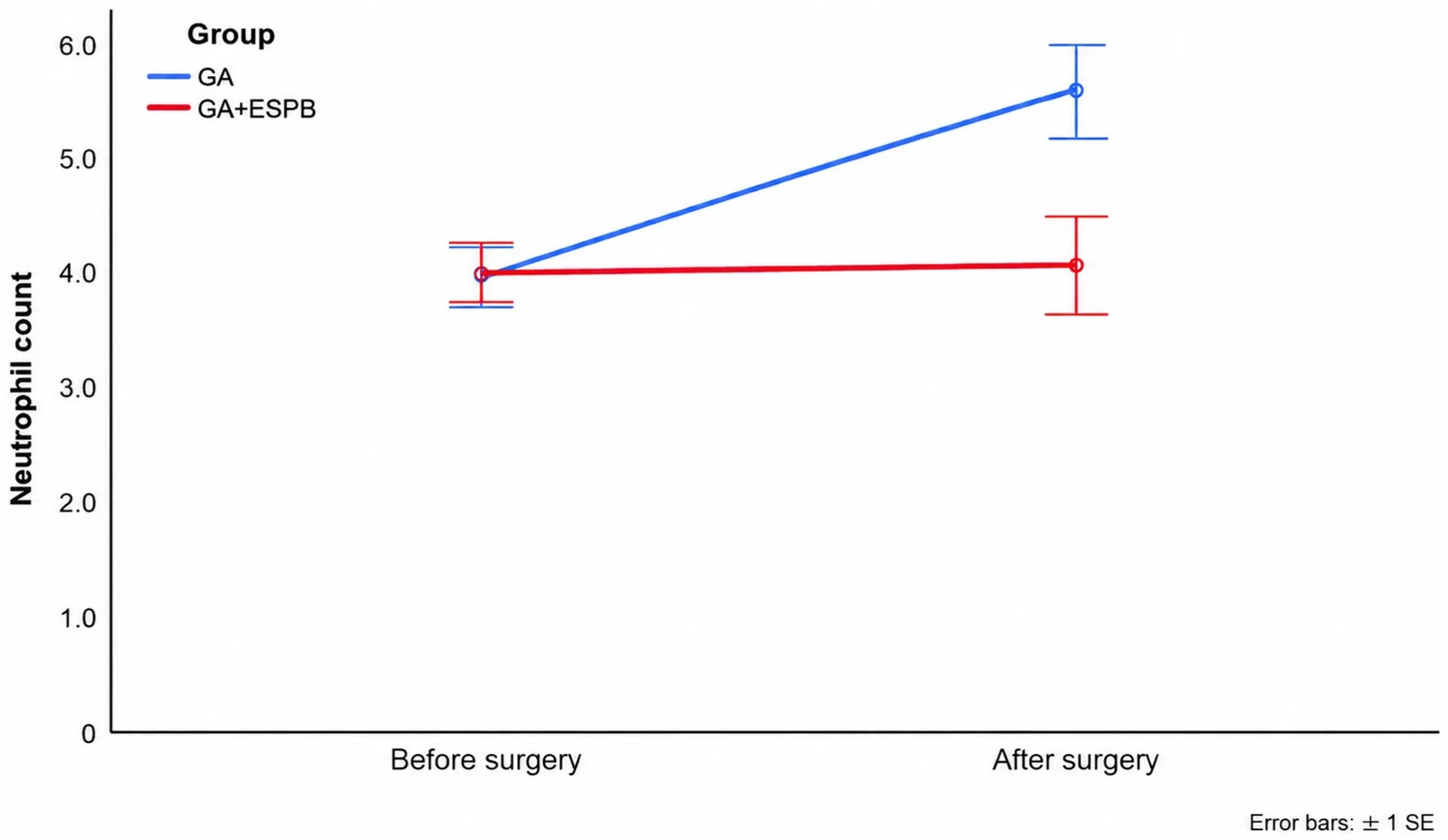
Neutrophil counts before surgery and during the immediate postoperative period in the GA and GA + ESPB groups.

**Figure 4:**
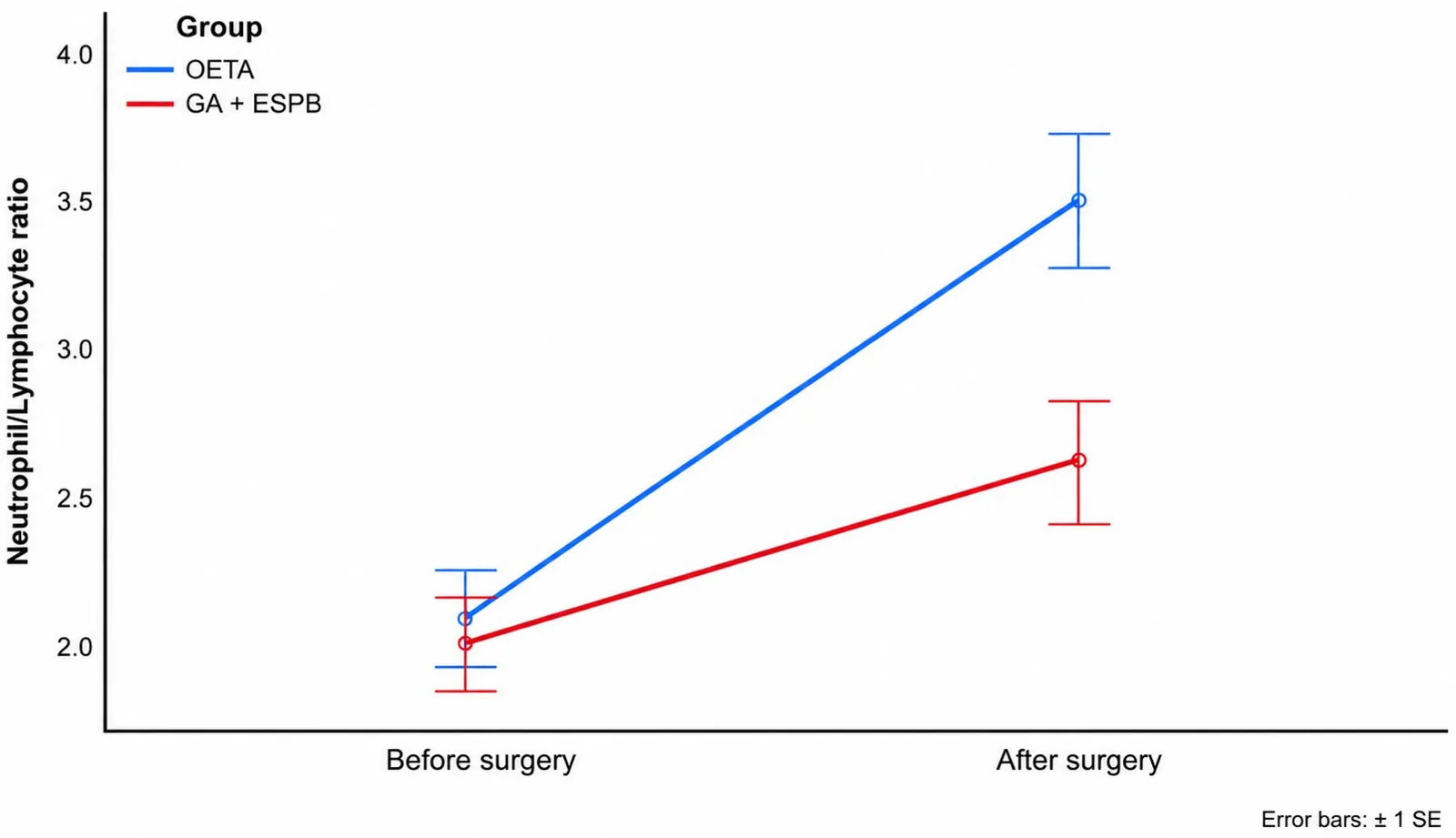
NLR values before surgery and during the immediate postoperative period in the GA and GA + ESPB groups.

**Figure 5:**
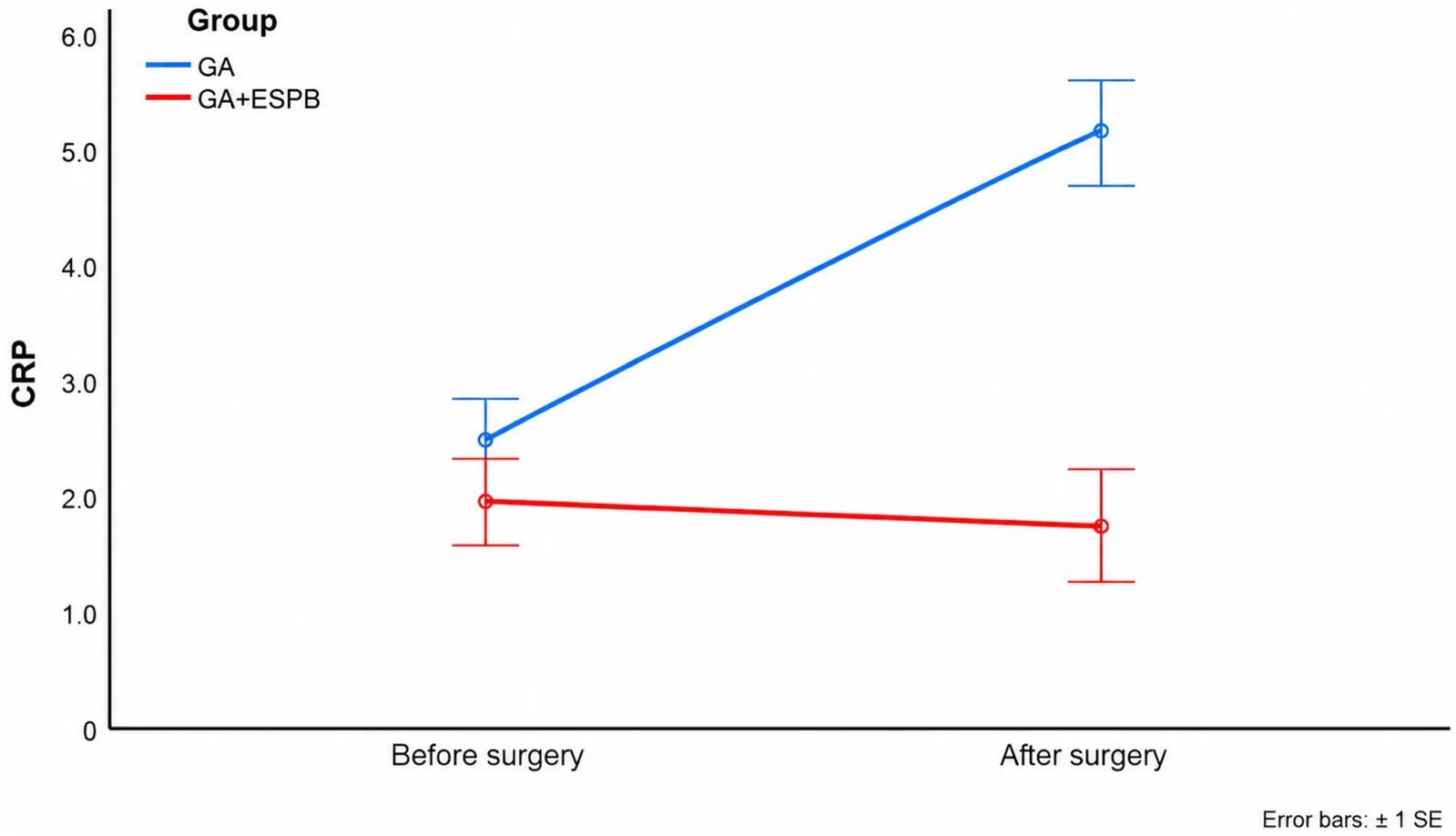
CRP levels before surgery and during the immediate postoperative period in the GA and GA + ESPB groups.

**Figure 6:**
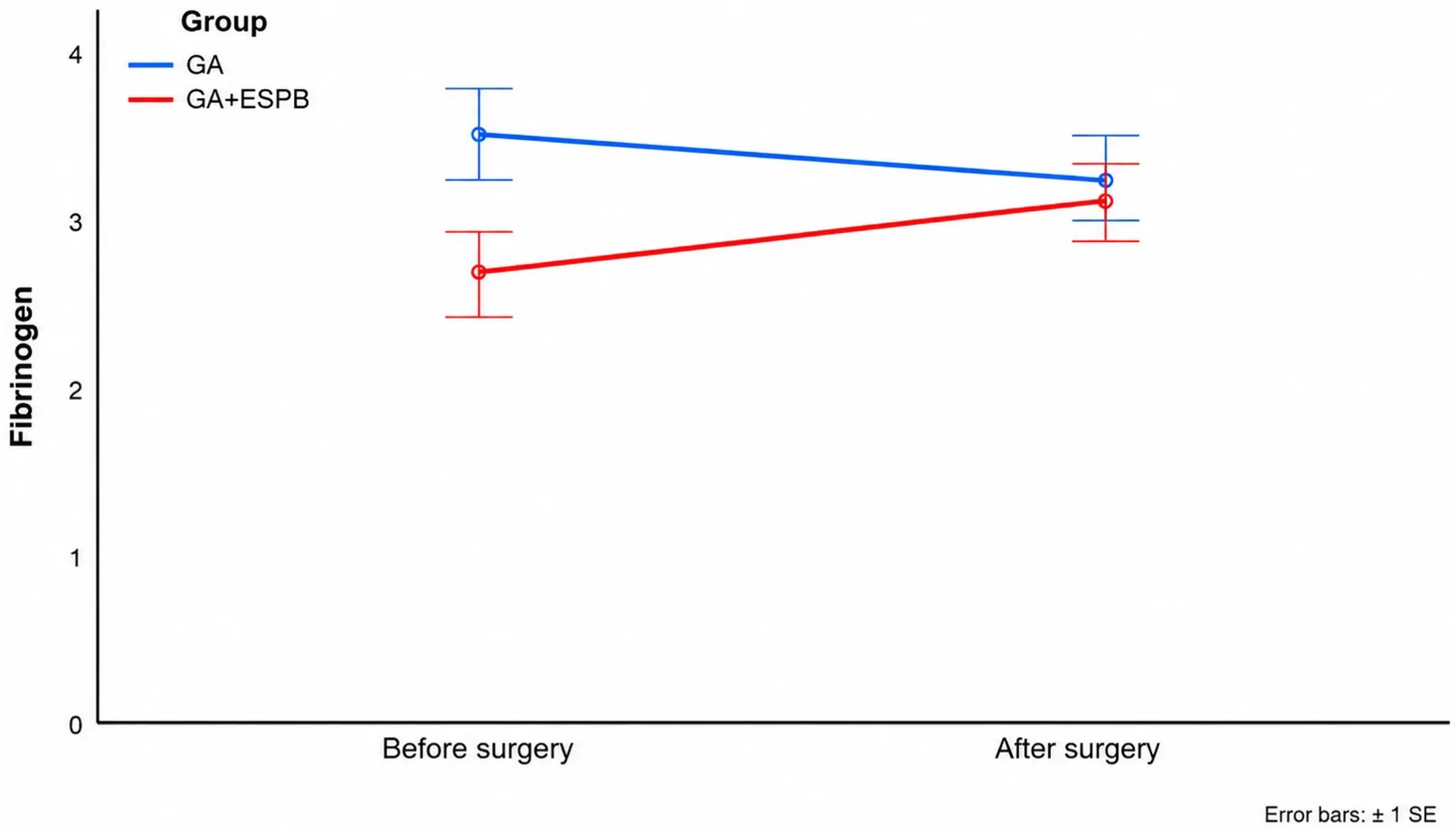
Fibrinogen levels before surgery and during the immediate postoperative period in the GA and GA + ESPB groups.

**Figure 7:**
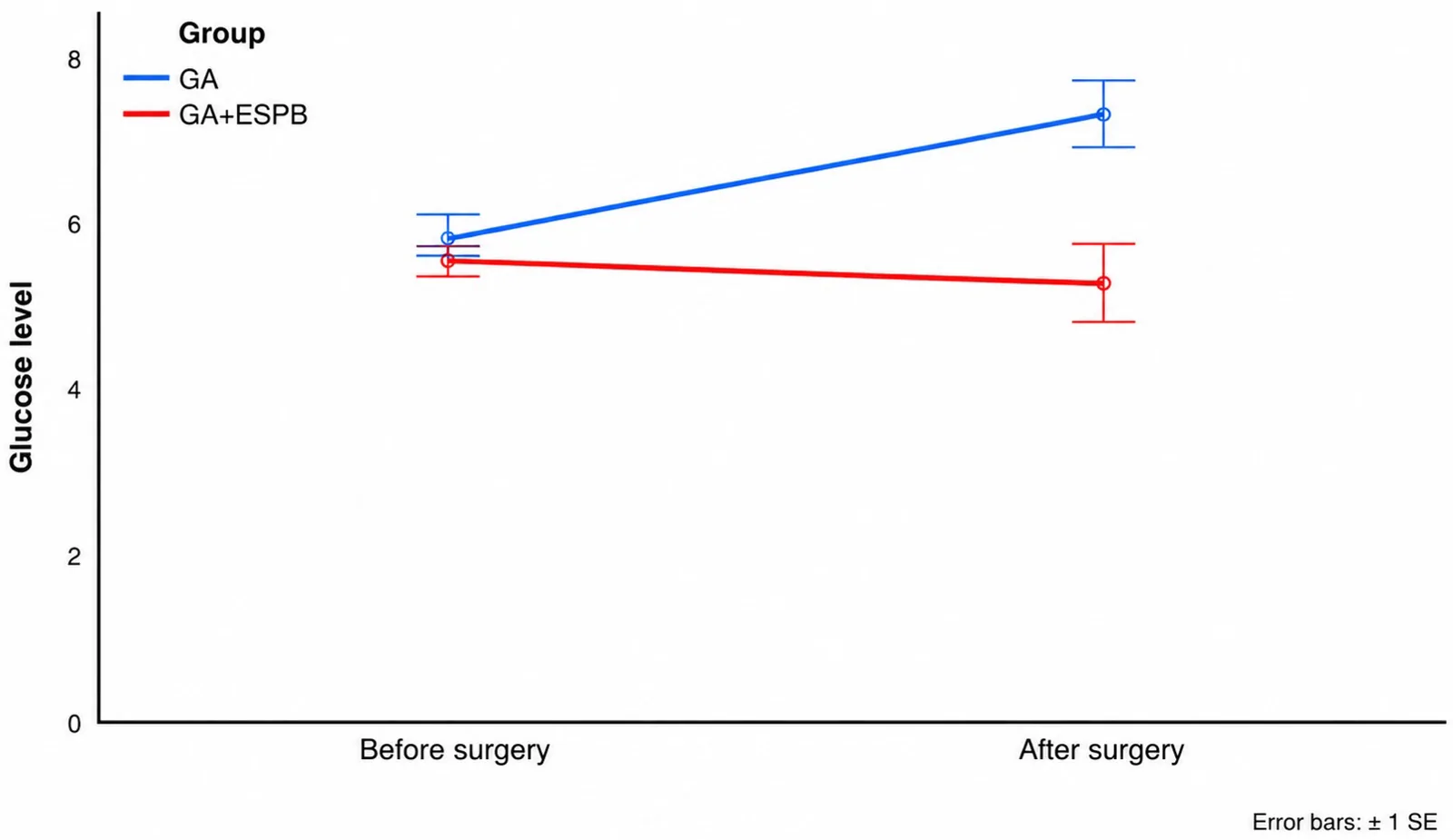
Blood glucose levels before surgery and during the immediate postoperative period in the GA and GA + ESPB groups.

**Table 1:** Baseline demographic and clinical characteristics of the study groups.

Characteristics	GA Group (*n* = 24)	GA + ESPB Group (*n* = 24)				*p* ^1^
Age (yr)	60.96 ± 5.51	58.50 ± 6.68				0.171
BMI (kg/m^2^)	24.54 ± 1.93	24.17 ± 1.49				0.456
ASA status						
I	*n*-%	12	50.0%	13	54.2%	1.000 ^2^
II	*n*-%	12	50.0%	11	45.8%	

^1^ *t*-test, ^2^ χ^2^ test.

## Data Availability

The datasets generated and/or analyzed during the current study are available from the corresponding author upon reasonable request.

## Abbreviations

The following abbreviations are used in this manuscript:

ASA	American Society of Anesthesiologists
CRP	C-reactive protein
ECLIA	Electrochemiluminescence immunoassay
ESM	Erector spinae muscle
ESPB	Erector spinae plane block
GA	General anesthesia
IFCC	International Federation of Clinical Chemistry and Laboratory Medicine
IL-6	Interleukin-6
MRM	Modified radical mastectomy
NLR	Neutrophil-to-lymphocyte ratio
PECS	Pectoral nerve block
TCI	Target-controlled infusion
